# Emerging Adults’ Experiences of Digital Measurement-Based Care in Routine Mental Health Care: Qualitative Study and Conceptual Synthesis

**DOI:** 10.2196/96322

**Published:** 2026-09-11

**Authors:** Geneca Henry, Courtney Habina, Marianne Barker, Emily Mair, Melissa Potestio, Tom Mogan, Julia Hews-Girard, Katherine Bright, Haley M LaMonica, Ian B Hickie, Frank Iorfino, Paul D Arnold, Gina Dimitropoulos

**Affiliations:** 1 Mathison Centre for Mental Health Research and Education, University of Calgary, Calgary, AB, Canada; 2 Hotchkiss Brain Institute, University of Calgary, Calgary, AB, Canada; 3 Department of Psychiatry, Cumming School of Medicine, University of Calgary, Calgary, AB, Canada; 4 Faculty of Social Work, University of Calgary, 3280 Hospital Drive NW, Calgary, AB, T2N 4Z6, Canada, 1 402-210-6464; 5 Recovery Alberta, Calgary, AB, Canada; 6 Faculty of Nursing, University of Calgary, Calgary, AB, Canada; 7 Alberta Children’s Hospital Research Institute, University of Calgary, Calgary, AB, Canada; 8 School of Nursing and Midwifery, Faculty of Health, Community, and Education, Mount Royal University, Calgary, AB, Canada; 9 Brain and Mind Centre, The University of Sydney, Sydney, New South Wales, Australia

**Keywords:** emerging adults, mental health, digital measurement-based care, patient-reported outcome measures, qualitative research, thematic analysis

## Abstract

**Background:**

Emerging adults (aged 18‐29 years) experience a high burden of mental health (MH) concerns during a developmental period marked by increasing autonomy and vulnerability to symptom onset. Many emerging adults face challenges in accessing and sustaining engagement with developmentally appropriate care. Digital measurement-based care (dMBC), which involves the routine use of patient-reported outcome measures (PROMs), has been introduced as an approach to support emerging adult engagement and improve responsiveness of care through ongoing monitoring and informed clinical decision-making. However, its integration into routine care remains variable, and there is limited understanding of how emerging adults experience and engage with dMBC in practice.

**Objective:**

This study aimed to examine how emerging adults experience dMBC within routine MH care and identify factors relevant to its use and integration in clinical practice.

**Methods:**

Using a descriptive qualitative design, semistructured interviews were conducted with 23 purposively sampled emerging adults who were either actively receiving care or had recently been discharged from 1 of 2 outpatient MH clinics in southern Alberta, Canada. dMBC was delivered through a web-based platform that enabled emerging adults to complete PROMs and view their results, which were also available to clinicians to inform care. All participants completed PROMs at baseline, with subsequent completion varying across participants and over the course of care. Interview data were analyzed using thematic analysis.

**Results:**

Four interconnected themes were identified and synthesized into a higher-level conceptual representation of emerging adults’ experiences with dMBC: technology, tracking, translation, and therapeutic guidance. Technology captured participants’ varied experiences with accessing and using the digital platform, including its usability and accessibility. Tracking reflected how monitoring PROM results over time supported reflection on changes in MH. Translation described how PROM data were interpreted and incorporated into therapeutic discussions, while therapeutic guidance reflected the role of clinician involvement in supporting the use of dMBC in care. Across the themes, participants described variation in the relevance and burden of measurement and in how consistently dMBC was integrated into clinical care.

**Conclusions:**

Emerging adults’ experiences of dMBC were shaped by both their interactions with digital measurement and the integration of PROMs into routine MH care. The findings highlight the importance of considering dMBC within the broader therapeutic context in which measurement occurs, rather than as a stand-alone digital activity. The conceptual synthesis provides a way of organizing these experiences and can inform future research and implementation efforts focused on the use of dMBC in emerging adult MH services.

## Introduction

### Background

Emerging adults, aged 18 to 29 years, represent a distinct developmental population situated between late adolescence and full adulthood [1,2]. This period is characterized by increased autonomy in decision-making, transitions into postsecondary education or employment, and shifting relational and financial responsibilities, often accompanied by reduced external structure or support [2-4]. At the same time, emotional regulation, identity formation, and higher-order cognitive functioning continue to mature, creating a developmental context that can foster growth while also heightening vulnerability [2-4]. These intersecting developmental and social transitions contribute to increased risk for the onset or exacerbation of mental health (MH) and substance use concerns during this stage [5-9]. Epidemiological evidence consistently indicates that about 75% of lifetime MH disorders emerge before the age of 25 [10,11], a period also associated with early patterns of unmet need, delayed help-seeking, and premature disengagement from care [12,13]. Whether entering services for the first time through adult programming or navigating movement across child and adolescent (hereafter “youth”) and adult services, many emerging adults encounter systemic barriers. These include long wait times, inconsistent eligibility thresholds, financial strain, and stigma-related concerns, which can contribute to delayed access and early disengagement [14,15]. As a result, emerging adulthood is now a priority focus within MH research and service innovation, driven by the need to improve early identification, deliver developmentally appropriate care, and ensure continuity of engagement across service contexts.

### Digital Measurement-Based Care

To address persistent barriers faced by young individuals, MH service systems increasingly use digital tools to expand access and strengthen engagement in care [9,16,17]. This addition is particularly salient for emerging adults, who are among the most digitally connected age groups and frequently use online platforms in daily life. Digital measurement-based care (dMBC) leverages this by embedding routine collection of patient-reported outcome measures (PROMs) within web- or mobile-based platforms, supporting their use as an adjunct to clinical care and informing ongoing clinical decision-making through clinician-patient review [18,19]. PROMs are standardized, validated questionnaires (eg, the Kessler Psychological Distress Scale, the Columbia-Suicide Severity Rating Scale, and the Quick Inventory of Depressive Symptomatology) that allow individuals to self-report their symptoms, functioning, and overall well-being, providing direct insight into their experiences [20]. When collected routinely, symptom monitoring and feedback can enable timely clinical responses to evolving needs and support adaptive treatment planning [21,22]. Studies show that regularly reviewing PROM data increases the likelihood that clinicians detect symptom changes or a lack of improvement, particularly among individuals who are not progressing as expected [23].

Integrating dMBC into routine MH care goes beyond in-session encounters by encouraging young individuals to stay engaged and involved in their care between appointments [24]. Self-assessment outside scheduled visits supports ongoing reflection on symptoms and experiences, which can improve communication and inform clinical decision-making over time [24]. Among young people, access to longitudinal feedback has been associated with greater insight into symptom patterns, treatment goal-setting, and stronger perceptions of participation in care when results are presented clearly [25-28]. Meta-analytic studies further indicate that digital MH interventions are associated with reductions in depression, anxiety, and stress symptoms among youth and young adult populations [17,29,30], with more robust effects observed when digital tools are integrated with active clinician involvement rather than delivered independently [31].

Growing evidence indicates that the value of routine PROMs depends not only on collecting outcome data but on how those data are understood and used within clinical care [32,33]. Implementation research has identified key barriers and facilitators affecting feasibility and service provider uptake across youth and emerging adult service settings. Reported barriers include insufficient clinician training and competing demands, difficulties integrating measurement into existing workflows, concerns about the relevance of measures, and uncertainty about engaging young people with differing needs or readiness for digital care [5,28,34-36]. Facilitators include service providers’ perceptions that young people are motivated to monitor change in their MH and comfortable using digital approaches, along with measurement platforms that provide timely feedback that can be incorporated into existing care processes [5,28,34-36]. Although this literature provides important insights into implementing dMBC, much of the evidence comes from service provider perspectives or broader youth populations. Emerging research directly involving emerging adults suggests that engagement may be supported when measurement is personally relevant, and PROM data are reviewed collaboratively within care, whereas standardized measures that do not reflect individual goals or experiences may discourage continued use [26-28,32,33,37]. However, understanding remains limited about how emerging adults experience dMBC when it is implemented as part of routine MH care.

### This Study

Research on dMBC with emerging adults is increasing, mainly from Australia [19,32,33,38-40], with comparatively limited research situated within Canadian MH services. The need for evidence grounded in emerging adults’ experiences of dMBC is also relevant to broader priorities for strengthening emerging adult MH care in Canada. The Mental Health Commission of Canada [41] identifies four priorities: (1) recognizing emerging adulthood as a distinct developmental stage and organizing services according to need rather than age; (2) partnering with emerging adults as experts in their own care to inform outcomes and service delivery; (3) improving collaboration and integration across services to support continuity of MH care; and (4) strengthening evaluation, research, data collection, and knowledge exchange to advance emerging adult MH care. By focusing specifically on emerging adults and examining their firsthand experiences of dMBC within routine MH care, this study responds to priorities around developmentally informed care and including emerging adults’ perspectives in understanding how care is delivered. It also contributes Canadian empirical evidence on how dMBC is experienced when incorporated into existing MH services, providing formative knowledge relevant to its integration and adaptation in practice. Accordingly, this study aimed to identify and describe key aspects of emerging adults’ experiences of using and engaging with dMBC.

## Methods

### Study Design

This study is part of a broader, ongoing implementation initiative focused on advancing the use of dMBC within emerging adult MH care pathways [42]. The broader study is guided by the Rapid Learning Health System (RLHS) framework [43,44], which uses real-time evidence to inform ongoing improvements in dMBC [42]. Within this framework, data produced through routine service delivery are revisited at multiple points to inform adjustments as implementation unfolds [45,46]. This qualitative study reports findings from the first set of interviews conducted in 2024 with emerging adults following the implementation of dMBC in 2023. A descriptive qualitative approach [47] using thematic analysis [48,49] examined how emerging adults understood and engaged with dMBC in routine care. The analysis identified patterns in participants’ experiences that highlighted factors supporting or hindering dMBC integration into care. These insights inform RLHS design by providing practice-informed evidence to support ongoing implementation.

### Study Setting and Service Context

This study was conducted at 2 MH clinics (one providing specialized services and the other offering community-based care) in Southern Alberta operating within the provincially coordinated health care system. Both clinics provide moderate-to-intensive outpatient care for individuals requiring frequent clinical contact without daily supervision, and offer intake assessment, individual and group psychotherapy, and psychiatric consultation with structured referral pathways that support continuity across services.

Across participating sites, dMBC was delivered through InnoWell [18] (InnoWell Pty Ltd), an Australian web-based platform that contains clinical measures for 23 biopsychosocial health domains (eg, Overall Mental Health, Depressed Mood, and Suicidal Thoughts and Behaviors). The platform scores PROM data and displays results in a dashboard for each domain using graphical trendlines and color-coded severity indicators to support longitudinal monitoring. An embedded alert system flags elevated risk responses on measures related to self-harm and suicide-related behaviors to enable timely clinical review [50]. Each participating clinic adheres to its established protocols for monitoring and responding to crisis alerts. A comprehensive description of the measures included in these domains is available in the broader RLHS study’s research protocol [42], with additional details on the implementation and application of InnoWell reported in related publications by the research team [5,26-28,34,35,51,52]. This study is not platform-specific; rather, it examines dMBC more broadly as an approach to integrating PROMs within routine MH care.

Within participating clinics, dMBC collection and review were integrated into a clinically stage-informed stratified model of care [42]. This model provides the service context in which dMBC was implemented; however, this qualitative inquiry focused on emerging adults’ experiences with dMBC rather than their experiences with clinical staging or stratification. In this model, clinical staging categorizes individuals based on the progression and complexity of their MH presentation, and stratified care uses this categorization to match treatment intensity and service type to symptom severity, functional impact, and risk [18,19,39,53]. At both clinics, individuals first participated in a psychoeducational group prior to receiving any formal treatment. This group supported goal-setting and assessed readiness for treatment, while also informing clinical staging alongside intake data (which may include InnoWell data or other measures used by the clinic) and clinical observations. Individuals were allocated to low- or high-intensity treatment streams, with additional referrals to psychiatry, group therapy, addictions, or family services as needed. Once emerging adults were placed in treatment, clinicians could use PROM data, in conjunction with clinical judgment, to iteratively adjust treatment intensity and direction.

PROMs were administered at research-specified time points, including baseline (at intake or start of treatment), 6 months, 12 months, and discharge. At these time points, the research team notified emerging adults that PROMs were due, with up to 3 contact attempts made by email, text message, or telephone. PROMs could also be completed at additional time points determined by clinicians based on clinical need, typically at approximately 2‐ to 3-month intervals, or at the emerging adult’s discretion during treatment. For these additional completions, emerging adults accessed the platform directly without notification from the research team.

### Participants and Recruitment

Eligible participants were drawn from individuals who had previously consented to participate in the broader implementation study and had been onboarded to the dMBC platform as part of their care at a participating clinic. In the broader implementation study, participants enrolled were aged 17 to 25 years and were actively receiving services at one of the participating clinics. The upper age limit of 25 years applied across both clinics to ensure consistent eligibility criteria across sites, as one participating clinic served emerging adults up to 25 years of age, while the other served individuals across the broader adult age range. Youth aged 17 years who were close to turning 18 years were included because they were on the cusp of emerging adulthood and received the same services as eligible emerging adults. Given the small sample, these individuals were grouped with the emerging adult cohort.

As part of the broader study consent process, participants were informed about the qualitative interview component and the opportunity to participate in interviews about their experiences with dMBC. To be eligible for this study, participants also had to have completed PROMs at baseline, defined as completing at least 70% of the PROMs across the 23 domains administered through the platform. At any PROM administration, participants were not required to answer every question or domain and could skip items they found too emotionally sensitive, difficult to answer, or not relevant to their treatment and goals. Completion of subsequently administered PROMs after baseline was also not required for eligibility. This allowed the study to include participants with varying levels of PROM engagement beyond baseline and who may have been at different points in their care (eg, earlier in care or approaching discharge), with differing opportunities to complete additional PROMs. Including participants with limited or no PROM completion beyond baseline was particularly relevant for understanding experiences that may have contributed to lower or discontinued engagement with dMBC. Thus, the sampling approach was intended to capture a range of experiences with dMBC following baseline PROM administration.

A purposive sampling strategy [54] was used to recruit these eligible individuals. A member of the research team contacted these individuals via email, telephone, and/or text message to provide information about the qualitative study and to gauge their interest in participating in an individual semistructured interview about their experiences with dMBC. Those who expressed interest received further details and were scheduled for an interview at a mutually convenient time.

### Data Collection

Semistructured interviews were conducted virtually via the University of Calgary–licensed videoconferencing platform, Zoom (Zoom Communications, Inc) [55]. An experienced member of the qualitative research team conducted the interviews, which lasted approximately 60 minutes and explored participants’ experiences with the dMBC platform and its role in their care. All sessions were audio-recorded and transcribed verbatim using the transcription service, Rev (Rev.com, Inc). A member of the research team reviewed each transcript to confirm accuracy and remove any identifying information before analysis.

The research team developed a study-specific interview guide to examine participants’ experiences with dMBC, including implementation, engagement, therapeutic integration, and overall usability. The guide explored onboarding and initial impressions, experiences completing PROMs, clinician integration of platform data into care, perceived impacts on engagement and therapeutic processes, and barriers and facilitators to ongoing use. Questions were open-ended, neutrally framed, and organized into modular sections to accommodate participants’ varying levels of platform engagement. Demographic data (eg, age, gender, and ethnicity) were also collected through an online survey.

### Data Analysis

Data analysis followed Braun and Clarke’s foundational [48] and updated guidance [49] for thematic analysis. The analysts held qualitative research roles within the broader study and were therefore familiar with the broader implementation context; this positionality informed their analytic engagement. All anonymized transcripts were imported into the qualitative data analysis software, NVivo (Lumivero), to facilitate data management and coding. Analysis began with repeated reading of the transcripts to become familiar with the data. A few members of the research team independently generated initial inductive codes that captured participants’ accounts. Each analyst developed a preliminary analytic codebook, including code labels, definitions, and illustrative excerpts drawn directly from the data.

Following independent coding, the analysts compared interpretations and resolved differences. They consolidated their individual codebooks into a single codebook, collaboratively refined code definitions, and resolved overlaps. This consolidated codebook guided subsequent coding refinement, with iterative adjustments made as analysis progressed. Regular meetings supported continued discussion of analytic decisions and ensured shared understanding of emerging patterns. The team then organized codes into broader patterns of shared meaning and developed them into preliminary themes. The team reviewed these themes against the full dataset to ensure they accurately represented the data. Through team discussion, the team finalized the themes to ensure each conveyed a distinct analytic focus.

### Ethical Considerations

The Conjoint Health Research Ethics Board at the University of Calgary granted ethical approval for this study (REB21-0616). Consent and demographic information were collected digitally and stored in REDCap [56-58], a secure, institution-hosted platform that supported controlled data entry and structured data management. All participants received a CAD $25 e-gift card (approximately US $18, based on the exchange rate at the time of the study) in recognition of their time and participation.

Participants reviewed study information and provided written consent prior to enrollment in the study. For participants younger than 18 years of age, a member of the research team administered a brief 4-item decision-making questionnaire to assess capacity to consent. A score of 4/4 was required to classify a participant as a mature minor, allowing participation without parental consent. Participants who scored below 4/4 had the incorrectly answered items reviewed and reassessed, with up to 3 attempts permitted. Those who did not achieve a perfect score after 3 attempts were deemed ineligible, as they did not meet the criteria for mature minor status.

Protecting participant confidentiality remained central throughout the study. Information that could reveal treatment involvement, assessment timing, or placement within the treatment pathway is not reported. Demographic categories with fewer than 10 counts are masked to reduce reidentification risk, in accordance with the Government of Alberta’s guidelines on reporting aggregated health information [59].

## Results

### Participant Characteristics

A total of 22 emerging adults participated in the study. All participants provided demographic information except one individual, who declined to complete the survey. Detailed demographic results are not presented due to confidentiality considerations [59]. Although individuals aged 17 to 25 years were eligible for recruitment, the participants ultimately recruited ranged from 17 to 24 years of age, with the majority aged 18 years. The sample primarily consisted of individuals who identified as women; overall, participants did represent diverse sexual and gender identities. Most participants identified with European heritage and were either born in Canada or had lived in Canada for more than 5 years. Participants also represented a range of educational, employment, and living circumstances, including students and individuals not currently enrolled in school; employed or unemployed participants; and varied housing arrangements, such as living with parents or caregivers, as well as other living situations (eg, living alone or with roommates).

Most participants had completed PROMs beyond baseline assessment, although the frequency, timing, and completeness of PROM engagement varied. Participants were at different points in their course of treatment and therefore had varying opportunities to complete PROMs following baseline. Most participants completed PROMs beyond the research-specified time points, with the largest group completing them regularly during care (eg, biweekly or monthly) and others completing them approximately every 2‐3 months, as determined by their clinicians. A smaller proportion completed PROMs only at the research-specified time points or had only completed the baseline assessment. PROM completion also varied, as participants could skip individual questions or domains. Cross-group comparisons based on participant demographic characteristics or patterns of PROM completion were not conducted to minimize the risk of identifying participants through combinations of potentially distinguishing characteristics within the small sample. A forthcoming manuscript will provide a more detailed examination of PROM trends at the research-specified time points and patterns of engagement with the dMBC platform across the broader study.

### Thematic Findings

#### Overview

**Figure 1.**
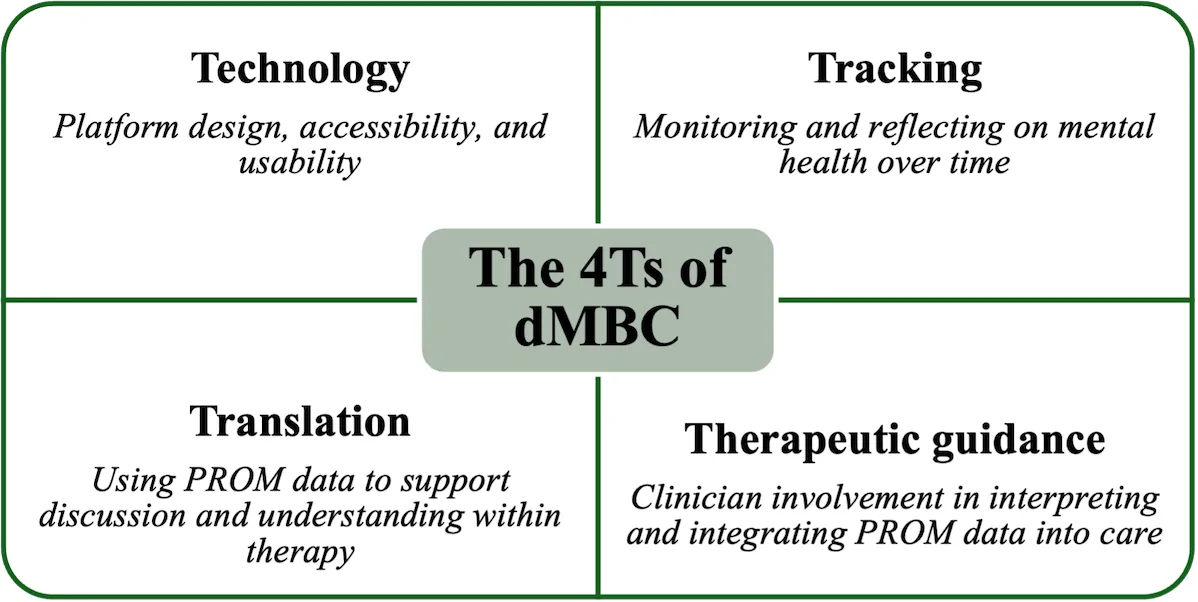
Conceptual representation of the thematic findings: the 4Ts framework. dMBC: digital measurement-based care; PROM: patient-reported outcome measure.

The thematic analysis identified 4 interconnected broad themes that characterized emerging adults’ experiences of and engagement with dMBC: technology, tracking, translation, and therapeutic guidance. These themes captured distinct but interrelated aspects of how dMBC was experienced within routine MH care and integrated into clinical conversations. To represent the findings at a higher conceptual level, the 4 themes were synthesized and organized as the “4Ts” framework (Figure 1). The framework is not presented as a separate analytic finding or exploratory model; rather, it serves as a conceptual representation that organizes and synthesizes the 4 broad themes identified through the analysis. Within this representation, technology reflects how digital accessibility and usability shape engagement with dMBC. Tracking captures emerging adults’ experiences of monitoring their MH and developing self-insight through repeated measurement. Translation reflects the use of PROM results to support the articulation of MH experiences and facilitate therapeutic dialogue. Therapeutic guidance captures clinicians’ role in reviewing, interpreting, and integrating PROM data within ongoing care.

The following thematic presentation illustrates how these interconnected aspects of emerging adults’ experiences with dMBC were shaped by the context of everyday care. Rather than reflecting isolated facilitators and barriers, the findings demonstrate how experiences across the 4 themes intersected to shape engagement with dMBC and the meaningful use of PROM data in clinical care.

#### Technology

Participants described varied experiences of accessing and using dMBC in relation to the platform’s design. Many participants valued the ability to complete assessments independently and review results at their own pace, highlighting the platform’s user-driven nature. Visual elements, including the color-coded indicators and graph displays, were consistently described as facilitating the interpretation of MH changes over time. As one participant shared, “The colour coding helps me decipher what’s important at the time,” while another explained, “It was mostly the graphs [...] saying this has declined from last time or this has gotten better.” Others described the platform as intuitive and easy to follow, noting, “It’s pretty easy to navigate around [...] it’s not like the website was hard to follow,” and appreciated the clarity of the measures, with one remarking, “I couldn’t ask for better [PROMs] [...] it was phrased so well that I didn’t have to struggle with any of the questions.” A few participants highlighted the breadth of the content, stating, “It encompasses everything [...] it literally encompasses the entire [aspects of] mental health.” Moreover, the ability to engage at their own pace was a particular strength for some, such as the participant who noted, “I like that it’s completely user-driven [...] I like that I can go at my own pace.” However, some participants differed in how accessible and easy to use they found the platform’s design. Some participants described the platform as initially overwhelming or less suited to their individual needs, including one emerging adult who characterized the interface as “too doctory” and “not very neurodivergent friendly.” Another reported difficulty reading the platform because of “very light writing on light background,” illustrating how design features that were accessible to some participants could create barriers for others.

Despite these strengths, several usability challenges disrupted sustained engagement. Common issues included difficulties logging in or accessing the website were common, with one explaining, “I tried to sign into it [...], but it was hard to find the website to sign in and [...] access it.” Others described ongoing navigation concerns, such as “If I wanted to go and find specific resources [...] I’m not too sure how to get there again,” or experiencing technical issues, noting, “It would just be freezing [get] and stuck loading.” Participants also described features they felt would make it easier to access and return to the platform. Many highlighted the absence of a mobile app as a barrier to consistent use, remarking, “I feel like if it were an app that can be easy to just click and find, that would make it better.” Several also expressed wanting a reminder or notification feature to address forgetting to recomplete PROMs when they were not prompted to do so.

Participants’ experiences of the platform were also shaped by how the digital PROMs were structured and presented. While some found the PROMs clear and easy to complete, others described the number of questions as daunting, with one participant recalling, “I signed up, I looked at it, and then I was like, ‘Oh my God, there’s so [many] questionnaires.’” Some participants found certain questions repetitive or difficult to answer due to their wording or response scales, while others reported that the assessments took longer than expected. A smaller number described the emotional demands of completing measures, with one participant explaining that repeatedly completing questionnaires was “draining both physically and mentally,” while another noted that “a few of the questions were somewhat triggering.” These experiences showed variation in how participants engaged with the format and demands of digital measurement, alongside the differences they described in navigating and accessing the platform.

Training and onboarding emerged as an additional factor influencing engagement. Although some participants found the platform intuitive, others felt underprepared after onboarding and were uncertain about how to access specific areas or make full use of the features: “I knew the basics, but I didn’t really know lots of details or anything.” Several emerging adults expressed a need for ongoing, flexible training resources, particularly formats they could revisit independently, such as instructional videos, to support continued engagement and confidence in using the platform. One suggested, “[Training] videos might be a cool idea. Then I would be able to go back and check things and see where they are.” Participants differed in the amount of support they felt they needed. While some reported understanding it “right away” and knowing “exactly what I was doing,” others understood its general purpose but were “not sure exactly how to use it and which button did what.” Some participants wanted additional guidance, including one who felt “it would’ve been nice to have another person who knew the [platform] with me while I did my [PROMs the] first time around, such as their clinician.

#### Tracking

Tracking MH symptoms over time was described as a way for participants to reflect on their experiences and changes during care. This functionality supported greater awareness by enabling individuals to monitor changes, recognize patterns, and reflect on their progress. As one emerging adult explained, “The thing I like most about the platform is that I could track my progress.” While another noted, “It was nice just knowing where I am or where I was, so I can look back on it to see if things improved or if things declined.” Participants also valued the detailed visual summaries, with one noting that it “shows you all of your previous answers [...] it’s very detailed tracking,” which helped them interpret their progress. For some, seeing results offered another way of visually understanding their experiences, with one participant describing how “It gave me a better example of how I was doing internally.” Tracking also helped participants identify areas to focus on in treatment. Several described reviewing their PROM results to determine what they might raise in upcoming sessions, with one sharing, “I like how it tracks data, and then you could see what areas would need more work with the therapist.”

For individuals waiting to be assigned a clinician, access to the tracking tools provided an interim sense of support: “It’s good that they have a good resource for people in the meantime so that they still have the support they need.” Some participants also saw the benefit in continuing to track their MH outside of formal care, describing ongoing monitoring as personally useful even after discharge: “Even if it’s not being brought up with a therapist, I feel like it can still be used in my own personal time.”

Alongside these benefits, participants offered suggestions to strengthen engagement with tracking features. Several suggested that customizable reminders would help them maintain consistent use, with one noting, “It would be helpful if there was some sort of system [feature] that could allow the user to choose when they would like to receive a notification.”

#### Translation

Participants described how the platform could serve as a bridge between their internal experiences and clinical dialogue. Having their PROM results reviewed in session often helped initiate discussion and provided a shared reference point for emerging adults and clinicians. One emerging adult explained, “[My clinician will] bring up, ‘Hey, I saw this on your [results], let’s talk about it.” Others noted that the visual summaries made it easier to articulate concerns they might otherwise struggle to express: “Sometimes it’s hard to talk about how you’re feeling [...] so if it’s there, they can see that.”Some participants similarly valued knowing that their responses were visible to their clinician, describing this as a “two-way street” because it’s not up to me all the time to reach out [...] it’s there for my clinician to see.”

The platform also prompted moments of self-reflection that shaped what participants chose to discuss in therapy. For some, the results surfaced concerns they had not fully recognized: “It just brings things to light that I didn’t even think about at all until I get to the end of any specific section [...] and go, ‘Huh, that’s a little bit further in the red zone than I thought it would be.” These insights helped participants better understand their symptoms and informed the conversations they initiated with clinicians. However, participants did not always feel that the results accurately reflected their experiences. One emerging adult described repeatedly receiving a low score in the Resilience domain that “wasn’t really the case for my situation in my life,” while another questioned whether their score related to anxiety aligned with “the things that I was feeling.” Some also described domains as less relevant to their circumstances or felt that aspects of their MH were not adequately represented. One participant explained, “I have a lot more than depression and anxiety [...] so it couldn’t delve into too much on that area.”

Using the platform in sessions also appeared to strengthen participants’ sense of agency. Several emerging adults felt that engaging with their PROM results enabled a more active role in treatment planning, sharing that it “helped [me] have more of a say; my voice is definitely a bit louder.” Others emphasized that dMBC made sessions feel more interactive and collaborative: “Well, it felt much more interactive than, say, other medical visits I’ve ever had […] I can actually tell them and have already told them before walking into the session what’s actually going on with me, and it’s nice.”

When PROM results were routinely incorporated into sessions, some emerging adults experienced increased accountability and follow-through: “If I filled out the [PROMs], then [for] every therapy [session], it would be a goal for myself to fill it out so [my clinician] would know where I was at.” For some, this process led to concrete changes in their care trajectory, such as referral to additional services: “I was able to get connected with an addiction counsellor [...] and now we’re going through the process of me possibly going to a treatment centre.”

Participants also highlighted how the platform facilitated a focus on treatment. Reviewing results together with the clinician helped identify concerns that warranted attention: “Here are the things you need to work on; here are the things you’ve gotten better with.” Several appreciated that clinicians could view their responses in advance, noting, “It saves a lot of time [...] [my clinician] can just know [the focus] before I come in.” Despite these benefits, several emerging adults noted they sometimes lacked clarity on how to use their PROM results to guide conversations during sessions. Some were unsure about their role in initiating PROM-based discussions, with one participant explaining, “I didn’t really know if I was supposed to bring it up or if [my clinician] would bring it up,” which left them unsure how to translate their results into session topics. Others described being aware of changes in their results but not always knowing how to frame them in conversation, such as “I saw things go up or down, but I wasn’t sure how to talk about it in therapy.” These uncertainties were especially relevant when participants felt that scores or domains did not reflect their own understanding of their experiences, highlighting the importance of opportunities to clarify or contextualize results during clinical discussions. For example, one emerging adult reflected, “I was wondering whether I was actually experiencing signs of anxiety because sometimes it felt like it didn’t quite line up with the things I was feeling.”

#### Therapeutic Guidance

Clinician involvement was central to how participants experienced dMBC within care. As one emerging adult shared, “It’s nice when my therapist goes through it with me [...] it helps me understand what the numbers actually mean.” Another participant similarly described that reviewing results together helped them identify priorities, sharing, “When we talked about the scores, it made it easier to figure out what to work on next.” Participants also valued the opportunity to interpret results with someone who could provide clinical context. One emerging adult explained, “It is really good to have someone go through it with that’s like a professional to like, ‘Hey maybe this is a problem.’” Emerging adults also described how clinician engagement increased their motivation to continue tracking their MH. Several noted that when their clinician showed interest in their PROM results, it encouraged greater follow-through: “If [my clinician] was excited about it, then I felt more excited to keep doing it.” One participant similarly reflected, “I think with my [clinician] pushing me to do the InnoWell stuff, I don’t think I would’ve done it without my [clinician] for sure.”

For some, clinician involvement was not just beneficial but necessary for continued engagement. Several emerging adults reported that they were unlikely to use the platform independently, with one stating bluntly, “I don’t really use it unless we’re talking about it in session.” Others felt they would only recommend the platform to peers if used alongside a clinician, noting that “it works best when the therapist actually looks at it with you.” One participant explained that they would “only recommend” the platform to a friend if they had a therapist and would not recommend it as a stand-alone MH app. Participants whose clinicians did not integrate the platform into care consistently expressed a desire for more involvement, with one remarking, “I wish my therapist used it more because I think it would help guide our conversations.” Another commented, “I do wish that there was a bit more of an initiative with my clinic to be like, ‘Let’s look at your results,” while another reflected that their clinician “didn’t find it super important to [...] constantly be updating it, I guess.” Some participants also described instances where clinicians checked whether PROMs had been completed without incorporating the results into the session itself, such as “Are you taking your InnoWell survey?”

For many, the primary barrier to consistent use was forgetting. Participants emphasized the importance of clinician reminders and follow-up, with one sharing, “Honestly, I just forget about it unless someone reminds me.” Emerging adults described that when clinicians prompted them to complete the PROMs or reviewed results regularly, it reinforced that the platform had a clear role in their care. Some recalled being encouraged directly in session, “Hey, you haven’t done this in a while, let’s do [a] full [battery],” or having clinicians who “told me to do the InnoWell [measures]” as part of treatment. Others noted that seeing their clinician consistently referenced their PROM data made continued use feel purposeful: “[My clinician] usually looks at my [results] before I come in [...] ‘something’s changed here, let’s talk about that.’” One participant even reflected that knowing their clinician checked the platform “would [...] prompt me to use it more.” These findings show that clinician prompting could support continued use, while shaping expectations around when and how often PROMs were completed.

## Discussion

### Principal Findings

dMBC is increasingly recognized as an important component of youth and young adult MH services [19,53], yet its implementation remains variable [60,61] and may not adequately account for emerging adults’ needs as they transition across developmental and service contexts [5,32]. Findings from this qualitative descriptive study highlight that emerging adults’ engagement with dMBC is influenced by how digital measurement is embedded within the broader experience of care, including opportunities for self-reflection, communication with clinicians, and the clinical use of PROM data. An RLHS approach informed the examination of these experiences by emphasizing responsiveness to user perspectives and the ongoing adaptation of care processes. The analysis identified 4 interconnected themes: technology, tracking, translation, and therapeutic guidance, that describe the key aspects of how emerging adults encountered and used dMBC in practice. This 4Ts conceptual framework draws these themes into an overarching representation of the findings, highlighting how meaningful engagement with dMBC emerges through their interplay within routine care. The following discussion considers the significance of these findings in relation to the existing literature and their implications for integrating dMBC within emerging adult MH services.

### Technology: Experiences of Platform Usability and Digital Engagement

The findings highlight how the dMBC platform’s design and functionality shaped participants’ experiences of accessing and using digital measurement in routine clinical practice. Consistent with existing digital health research highlighting usability as a key factor in sustaining user engagement and adoption [26,62-65], this study indicates that the dMBC platform’s design and functionality played a central role in enabling the other components of the 4Ts model. When participants found the platform intuitive, visually clear, and reliable, they engaged with dMBC more effectively, both independently and collaboratively. In contrast, when usability challenges arose, they had a cascading effect, making consistent tracking difficult and limiting opportunities to meaningfully discuss PROM data during sessions and use the data to continue to inform treatment planning.

These findings also raise broader considerations for equitable engagement with dMBC, as technological barriers may not affect emerging adults equally. In a scoping review of ethical considerations surrounding digital MH technologies for young people, Wies et al [16] identified equitable access as a recurring concern, noting that socioeconomic circumstances and geographic location may contribute to a digital divide that disproportionately affects disadvantaged and rural populations. Another scoping review [66] of digital MH interventions for young people found that usability barriers could reduce engagement, while flexible and accessible intervention design supported continued use. Accessibility also depends on whether the mode of digital engagement responds to individual needs. For instance, neurodivergent individuals may experience greater difficulty filtering and prioritizing digital information, and repeated PROM completion and self-monitoring may create additional burden or distress for individuals vulnerable to anxiety or hypervigilance [67]. Considered alongside the present findings, this literature indicates that usability barriers may affect emerging adults differently depending on their access and accessibility needs. Flexible approaches and appropriate accommodations may therefore be needed when digital measurement does not adequately support an emerging adult’s engagement with care.

For sustained implementation, efforts to support clinician engagement or client motivation may be insufficient if they do not address technological barriers. Ongoing investment in user-centered design is therefore necessary to address structural barriers related to accessibility, usability, and developmental relevance [65,68,69]. This is particularly important given that youth and young adults primarily access digital content on smartphones rather than on desktop computers or laptops [70]. Platforms that are not optimized for mobile use may inadvertently limit engagement among the populations they aim to serve [26]. Digital MH tools also operate within an attention economy shaped by rapidly shifting, screen-based content, where frequent exposure to highly stimulating digital environments has been linked to reduced sustained attention and greater distraction in similar contexts [71-73]. Many young people also use social media platforms such as YouTube (Google LLC), TikTok (ByteDance Ltd), and Instagram (Meta Platforms, Inc) to seek MH information and make sense of their experiences [74]. Although these platforms can support help-seeking, they also introduce risks related to misinformation [74] and algorithm-driven amplification of oversimplified content [73]. The short-form, personalized, and highly immersive engagement strategies (eg, frequent updates, notifications, and gamification) common to commercial digital platforms further shape expectations about how digital content is accessed and experienced [73].

These broader digital expectations have implications for dMBC design, but engagement strategies used by commercial platforms may not translate directly to clinical contexts. Digital MH research has explored features such as gamification to support engagement; however, these features tend to be incorporated into therapeutic activities rather than used solely to sustain interaction [75]. Similarly, a recent scoping review [76] on digital MH intervention design and evaluation found that sustained engagement depended not only on features intended to motivate use but also on whether interventions were usable, accessible, and responsive to users’ needs and contexts. This highlights the importance of considering how engagement features affect the overall experience and demands of using a clinical platform. This is relevant to the present findings, as participants wanted more convenient access and reminders, while some also experienced repeated PROM completion as burdensome. Design features intended to support engagement should therefore complement the clinical purpose of measurement without increasing the demands of ongoing self-monitoring. Encouraging more frequent interaction with dMBC also raises broader considerations about privacy, data governance, and users’ understanding of how their information is collected and used [16,26], as ongoing measurement generates more personal MH data. Although participants did not explicitly raise concerns about data surveillance, these considerations are relevant when determining how engagement with dMBC should be supported. Similarly, the findings suggest that encouraging continued monitoring should be balanced against the risk that repeated measurement could lead to premature conclusions about symptoms through self-diagnosis. For dMBC to remain relevant in this broader digital landscape, its design should reflect the digital modalities and interaction patterns young people prefer, while maintaining clinical rigor [26,65]. Future research should examine how these design and data practices shape emerging adults’ experiences of digital measurement and trust in dMBC.

### Tracking: PROM Data for Reflection and Understanding Change

The study findings suggest that tracking provided participants with a way to reflect on changes in their MH and better understand their experiences over time. Longitudinal PROM collection prompted participants to pause and evaluate their mood, stress levels, and functioning at specific time points, which several participants described as increasing their awareness of fluctuations that might otherwise have gone unnoticed. This observation is consistent with research on self-monitoring, which shows that structured symptom reporting can heighten emotional self-awareness and improve recognition of mood variability over time [26,33,77,78]. The visual display of scores over time further supported this process by making shifts observable, reinforcing pattern recognition, and facilitating more accurate self-appraisal [26,32,33]. In this sense, tracking functioned as an embedded reflective exercise that strengthened emotional awareness by allowing individuals to repeatedly assess and compare their experiences over time, rather than serving merely as a passive source of clinical data.

The findings also suggest that the value of tracking may go beyond symptom recording, as making changes in MH visible over time provided participants with a meaningful basis for continued engagement with measurement. The visualization of progress reinforced engagement and readiness to remain involved in care and is consistent with evidence that structured feedback and graphical outcome displays can strengthen ongoing involvement in treatment [26,32,33]. When symptom trajectories are visible, individuals may be better able to connect effort with observable movement, a process that has been associated with enhanced self-efficacy and more sustained behavior change [32]. Alongside this, tracking bolstered a sense of autonomy and personal empowerment by positioning emerging adults as active interpreters of their own data [79-81]. Participants reviewed patterns, identified areas needing attention, and decided what to address in sessions, reflecting empowerment and essential self-regulatory processes of monitoring and evaluation in routine clinical care [78,82]. Digital monitoring tools can scaffold these processes by structuring reflection and supporting more deliberate coping responses (eg, self-management techniques such as emotional regulation strategies, cognitive restructuring, and behavioral modifications) [32,33]. These effects may be particularly salient for emerging adults who demonstrate greater readiness for change and intrinsic motivation to engage in recovery efforts related to MH challenges [5]. For those awaiting clinician assignment, continued access to tracking features helped maintain continuity of care. Digital approaches may offer additional ways for individuals to remain engaged with their MH care during periods when direct clinical support is limited [19]. In this context, tracking appeared to help sustain a sense of continuity and connection to care, even when direct contact with a clinician was temporarily limited. These findings suggest that tracking may function as an active therapeutic element within dMBC, rather than merely a procedural requirement for data collection.

Long-standing barriers to consistent symptom monitoring within MBC provide important context for the variability in platform use observed in this study [5,32,61]. Drawing on findings from Bassi et al [5], sustained self-report completion may be challenging for some emerging adults, particularly those living with certain psychiatric and neurodevelopmental conditions, which can shape how feasible or appropriate frequent PROM completion feels in daily life. For example, in that study, clinicians highlighted that youth and emerging adults living with these conditions, such as learning disabilities, autism spectrum disorder, attention-deficit and hyperactivity disorder, and enduring MH conditions such as bipolar disorder and schizophrenia, may find routine self-assessment cognitively demanding or misaligned with fluctuating insight, may have difficulty accurately self-assessing MH status, symptoms, or level of functioning, or may exaggerate or underreport or overreport concerns. The same study also found that lower motivation to engage with dMBC may reflect reluctance to complete therapeutic “homework” outside of sessions. Similarly, Chong et al [32] reported that participants experienced guilt for not completing measures, which at times intensified distress and further reduced engagement with symptom tracking. However, these participants reported disengagement during periods of low mood or hopelessness, when they perceived minimal improvement in scores.

### Translation: The Process of Translating PROM Data Into Therapeutic Dialogue

Translation reflected how participants discussed and interpreted insights gained through tracking within therapeutic dialogue. The findings suggest that PROMs supported dialogue by providing a shared language to articulate and discuss internal experiences more clearly [78]. Rather than relying on broad or imprecise statements of distress, participants could reference specific domains or score changes as anchors for discussion. This may be particularly important given that young people often use inconsistent or nonclinical language to describe their MH experiences and may experience uncertainty in how to conceptualize or communicate distress [83]. Structured PROM domains can help bridge this gap by linking subjective experiences to well-defined constructs, thereby supporting clearer communication between clients and clinicians. Research on feedback-informed treatment similarly suggests that routine outcome data can help identify concerns for discussion and inform treatment priorities [78,84,85]. For youth and emerging adults, structured measurement may therefore support better articulation of experiences that may be difficult to communicate [32].

However, the structure provided by PROMs also warrants consideration of how lived or living experiences are represented within care. Standardized measures define the domains assessed and the response options through which experiences are reported, meaning concerns outside these parameters may be less readily captured [32,78]. Dey et al [86] similarly highlight the importance of considering how standardized measurement reflects the complexity of individual experiences, while Poulsen et al [79] emphasize preserving autonomy and the young person’s role in determining what is meaningful within digitally supported MH care. This study’s findings suggest that structured PROM data are most useful when considered alongside emerging adults’ own accounts of their experiences. When scores or predefined categories take precedence, therapeutic discussions may focus on what the measures capture rather than on the concerns emerging adults identify as most relevant to their care.

The findings also indicate that access to PROM results did not necessarily give emerging adults a clear way to use that data in therapy. Some participants were uncertain about how to interpret changes in their results or whether they or their clinician should initiate discussion of them. This suggests that PROMs’ capacity to support emerging adult engagement depends, in part, on how results are introduced and interpreted within the therapeutic dialogue. Without this process, dMBC may be interpreted without sufficient clinical or personal context, potentially contributing to premature self-diagnosis [5] or misinterpretation of data [32]. Effective PROM-informed conversations, therefore, depend on open communication and collaborative interpretation, in which quantified data are examined alongside the emerging adult’s account of their experiences [33,81,87-89]. Translation can be understood as a relational interpretive process through which quantified information is discussed and situated within the emerging adult’s lived or living experience. This process supports the clinical use of PROM data while keeping the emerging adult’s perspective central to their interpretation and application in care.

### Therapeutic Guidance: Integrating dMBC Into Ongoing Care

Therapeutic guidance reflects how that meaning is subsequently integrated into clinical decision-making. The findings suggest that therapeutic guidance serves as the mechanism through which PROM data move beyond static information, becoming an active resource that informs treatment direction and supports sustained engagement in care. This conceptualization is consistent with long-standing evidence that clinician involvement is not merely an adjunct but essential for the effective use of measurements in MH treatment [78,90]. Across the study, participants consistently relied on their clinicians to help interpret scores, develop goals and priorities, and contextualize changes within their broader life circumstances. This reinforces research showing that PROMs have the greatest impact when incorporated into shared interpretation and collaborative planning [32,33,81,87-89]. The manner in which clinicians framed the results shaped whether participants perceived the data as useful, relevant, or concerning; when clinicians expressed enthusiasm and actively referenced PROM scores in session, it created a supportive, reinforcing loop that increased participants’ motivation to complete measures, track their progress, and remain engaged in care [32,33,78]. This dynamic reflects broader evidence highlighting therapeutic alliance and clinician responsiveness as critical predictors of whether feedback becomes actionable in practice [32,33,60,78,90,91]. Evidence further suggests that the identification of safety risks and suicide assessment is most effective within MH care contexts where a strong therapeutic alliance supports the interpretation of screening results, particularly given that broad population (eg, primary care clients) screening often produces false positives [91,92].

Conversely, when clinicians did not integrate PROMs into sessions, were inconsistent in doing so, or signaled ambivalence about their value to clients, participants perceived the platform as disconnected from their care, leading to diminished motivation and engagement and uncertainty about the purpose of completing data [61,93]. These findings indicate that without explicit clinician modeling and follow-through, dMBC risks becoming an administrative task rather than an integrated component of treatment and a way to facilitate and strengthen the client-therapist relationship [33]. This interpretation is also consistent with Dey et al [86], who found that patients may be uncertain about how their responses inform treatment decisions, highlighting the importance of making the clinical purpose and application of measurement clear. Therapeutic guidance thus supports moving PROM data from measurement to therapeutic insight and direction. PROMs should not operate independently; their value seemingly depends on how they are incorporated into the relational and clinical processes of care. This conceptualization reinforces dMBC implementation literature, emphasizing that digital tools require active clinician engagement to meaningfully influence care pathways and sustain user participation over time [33]. As Lewis et al [61] note, self-report data are most impactful when individuals understand the purpose of the measurement and see it as relevant and influential in decisions about their care. When that relevance is unclear, completion may decline, tracking can feel disconnected from lived experience, and distrust of the therapeutic relationship may ensue.

Clinicians’ central role in determining how PROM data are interpreted and acted upon also raises important questions about therapeutic authority. Clinician guidance can help emerging adults understand their results and connect them to their care, but it may also create expectations for continued PROM completion and place greater responsibility on emerging adults for ongoing measurement. Poulsen et al [79] distinguish empowerment from simply increasing an individual’s responsibility for self-monitoring, noting that expectations to collect and engage with health data can become a source of obligation or imposed control rather than empowerment. They further emphasize that autonomy involves not only opportunities for choice but also the ability to determine whether and how to engage with digital MH technologies. This distinction highlights the importance of considering whether clinician-supported engagement with dMBC preserves meaningful choice in how emerging adults participate in measurement.

This is relevant to the present findings, in which clinician interest, reminders, and follow-up increased participants’ motivation to complete PROMs and helped make measurement feel purposeful and connected to their care. Although participants did not explicitly describe feeling surveilled or pressured to report particular outcomes, increased completion should not necessarily be interpreted as greater agency or empowerment. The findings indicate that clinician involvement may be most meaningful when emerging adults understand the purpose of measurement and retain influence over how their results are interpreted and used within care. Clinicians should therefore encourage PROM engagement in ways that remain responsive to emerging adults’ preferences, including opportunities to contextualize or question their results and raise concerns that the measures may not capture. Repeated completion should not become an implicit expectation of care when the emerging adult no longer perceives measurement as meaningful or useful. Future research should examine how emerging adults experience expectations around repeated PROM completion, including whether ongoing self-monitoring feels supportive or burdensome and whether it creates pressure to demonstrate improvement.

### Strengths and Limitations

A strength of this study is the conceptual representation of emerging adults’ experiences, grounded in the thematic findings and consistent with the qualitative descriptive approach. The themes constitute the primary findings, with the 4Ts framework providing a higher-level synthesis that organizes their shared and interconnected features. This representation offers a practice-relevant way of considering factors that shape meaningful engagement with dMBC while remaining grounded in emerging adults within routine MH care. The 4Ts should not, however, be interpreted as a validated implementation model or exploratory theory. The relationships among the 4 themes were not empirically examined, and the framework was not developed to establish causal mechanisms or predict implementation outcomes. Further research is needed to examine its applicability across other service settings, care models, and populations, and determine its utility for informing dMBC implementation.

Moreover, the study drew on a modest sample from 2 similar clinical programs in a larger urban area, which may narrow the breadth of perspectives represented and may limit the extent to which the results apply to other service environments. The sample may also underrepresent emerging adults who experience barriers to digital engagement, including limited access to technology, lower digital literacy, or accessibility needs that affect their ability to use digital platforms or complete PROMs. The urban setting may also limit the representation of emerging adults living in rural or remote communities, where access to reliable internet, digital infrastructure, and in-person supports may differ. As a result, the findings may not fully capture how these barriers shape experiences of dMBC or contribute to inequities in its use. Future research should include emerging adults across a broader range of geographic and service contexts and consider how individual accessibility needs, including physical, emotional, or cognitive capacity that may require accommodation, shape experiences of and engagement with dMBC. This will help identify appropriate supports and inform more equitable approaches to dMBC implementation.

In addition, the analysis only centers on the experiences of emerging adults. This focus offers insight into user perspectives but does not reflect how clinicians, organizational leadership, and other varied perspectives within the service environment understand and experience dMBC. Research conducted within the broader study addresses these viewpoints through additional qualitative components. Although the scope of the present inquiry is bounded in these ways, the themes identified resonate with patterns observed in prior research on digital approaches to MBC, indicating that the issues raised here are relevant to settings with similar service structures and populations.

The study was also conducted within services using a clinically staged-informed stratified care model; however, clinical staging and stratification were not a focus of the qualitative inquiry. Participants were not specifically asked how PROM data informed staging processes or how they experienced measurement within this model. The findings therefore cannot speak to how this care model may have shaped emerging adults’ experiences of dMBC. However, this area was examined through subsequent qualitative interviews conducted with emerging adults in early 2025. Findings from this qualitative inquiry will be reported in a future publication.

### Next Steps

Consistent with the RLHS, findings from this study have informed dMBC implementation as the broader study has progressed. The research team shared findings with clinical partners, clinicians, and service leaders through knowledge translation activities to support ongoing implementation adaptations. These insights were considered alongside qualitative feedback collected from clinicians and clinical service leaders through focus groups, with findings from these interviews to be reported in future publications. Subsequent qualitative data collected from emerging adults, including their experiences of dMBC within the clinically stage-informed stratified care model, provided further evidence to inform the current phase of the broader study.

The current phase uses a user-centered, participatory co-design approach to develop and refine practice-oriented implementation resources for integrating dMBC into routine emerging adult MH care [94]. The co-design process engages emerging adults, clinicians, and clinical service leadership to identify feasible approaches that reflect the needs and experiences of those who use dMBC as part of their care, as well as those who deliver and support its implementation in clinical practice. This phase is not structured around the 4Ts framework or intended to test the framework. Rather, the findings and practice recommendations from this study, alongside other evidence generated through the broader study, informed the objectives and discussion prompts for the co-design sessions, which further explored implementation needs and potential solutions.

### Conclusions

This study provides insight into emerging adults’ experiences of dMBC within routine MH care and identifies considerations relevant to its integration into practice. The findings contribute to the emerging evidence base on dMBC from the perspective of those receiving care and have informed ongoing implementation learning within the broader RLHS study. In the Canadian context, this work advances national priorities for emerging adult MH care by generating formative evidence that recognizes emerging adults as contributors to understanding and improving care delivery.

## Abbreviations

dMBC: digital measurement-based care
MH: mental health
PROM: patient-reported outcome measure
RLHS: Rapid Learning Health System
